# Low temperature plasmas as emerging cancer therapeutics: the state of play and thoughts for the future

**DOI:** 10.1007/s13277-016-4911-7

**Published:** 2016-02-18

**Authors:** Adam M. Hirst, Fiona M. Frame, Manit Arya, Norman J. Maitland, Deborah O’Connell

**Affiliations:** 1 Department of Physics, York Plasma Institute, University of York, Heslington, UK; 2 YCR Cancer Research Unit, Department of Biology, University of York, Heslington, UK; 3 University College London, London, UK

**Keywords:** Low temperature plasma, Reactive species, Focal therapy, Cancer stem cells, Combination therapy

## Abstract

The field of plasma medicine has seen substantial advances over the last decade, with applications developed for bacterial sterilisation, wound healing and cancer treatment. Low temperature plasmas (LTPs) are particularly suited for medical purposes since they are operated in the laboratory at atmospheric pressure and room temperature, providing a rich source of reactive oxygen and nitrogen species (RONS). A great deal of research has been conducted into the role of reactive species in both the growth and treatment of cancer, where long-established radio- and chemo-therapies exploit their ability to induce potent cytopathic effects. In addition to producing a plethora of RONS, LTPs can also create strong electroporative fields. From an application perspective, it has been shown that LTPs can be applied precisely to a small target area. On this basis, LTPs have been proposed as a promising future strategy to accurately and effectively control and eradicate tumours. This review aims to evaluate the current state of the literature in the field of plasma oncology and highlight the potential for the use of LTPs in combination therapy. We also present novel data on the effect of LTPs on cancer stem cells, and speculatively outline how LTPs could circumvent treatment resistance encountered with existing therapeutics.

## Introduction

The role of reactive species in cancer initiation, progression and treatment has been intensively researched over the last few decades. The mechanistic actions of radio- and chemo-therapies frequently rely on the formation of reactive species, and they have been proposed as a means to preferentially target malignant cells [1]. Low temperature plasmas are known to generate a plethora of reactive oxygen and nitrogen species [2], and could present an exciting new modality for the treatment of tumours.

Plasmas are ionised gases, comprising a complex environment of charged particles, neutral gas molecules, UV radiation, electric fields and reactive species. They occur widely in nature (for example as lightning or the aurora borealis), yet can also be created in many forms in the laboratory to exploit their unique properties for many varied applications, from surface modification to clean energy production. Due to technological advancements, it has become possible to sustain plasmas at atmospheric pressure and room temperature. This has enabled the use of plasmas in a range of technological and biomedical applications, and thus the conception of the field of ‘plasma medicine’ over the last decade. ‘Low temperature plasmas’ (LTPs) are very weakly ionised; the electrons, which can have temperatures ∼10^4^ K and drive the plasma processes, make up a very small fraction of the plasma (<0.1 %). The bulk of the plasma consists mainly of background neutral gas atoms and molecules, and due to the inefficient energy transfer between the light electrons and ‘heavy’ neutrals, the global environment remains at room temperature. This aspect allows the application of LTPs to temperature-sensitive materials, such as living tissues.

**Fig. 1:**
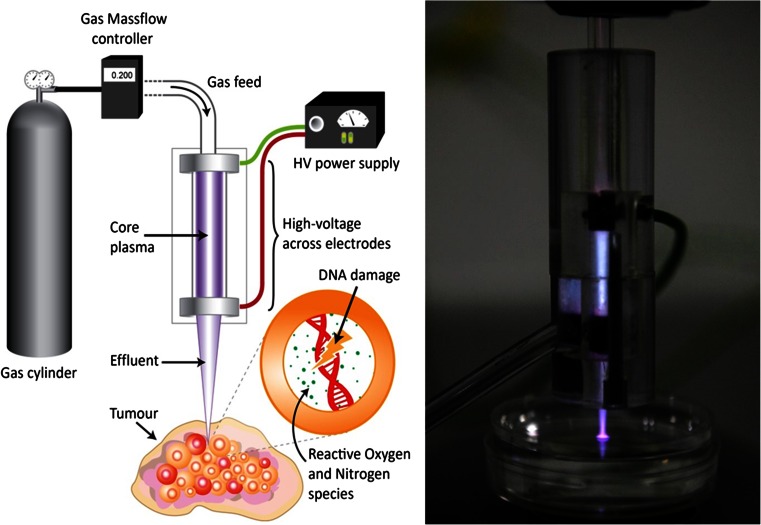
Schematic representation of low temperature plasma formation and application. Gas flow is ignited by high voltage applied across ring electrodes. The core plasma propagates from the end of the tube and is applied into a bulk tumour, causing DNA damage through the formation of reactive oxygen and nitrogen species. Note: this diagram is not to scale; in the accompanying image, the dimensions of central quartz glass tube are 70 × 6 mm. Elements of this figure are modified from Hirst et al. [6]

The general concept of plasmas in medicine is not totally new, as they have been utilised as electrosurgical instruments in medical practice for a number of years and in a range of procedures [3]. Recent innovations include instruments from Plasma Surgical and Arthrocare; hand-held devices capable of vaporising, sealing and dissecting tissues [4, 5]. LTPs are fundamentally different; as their name suggests, they do not utilise thermal effects to induce biological response. Instead, they induce biological response through the production of reactive species and potentially strong electric fields, and are a novel proposition for use in medical procedures. A sketch and photograph of a typical laboratory LTP jet set-up is shown in Fig. 1. Controlled gas flow is fed through glass tubing, around which high-voltage electrodes are positioned. The core plasma is ignited between these electrodes by applying a high voltage (typically up to 20 kV), and a plasma jet then propagates outwards and can interact with the biological sample. It is important to note that Fig. 1 represents only a single example; many different LTP designs and geometries exist that are intended for biomedical applications [7–11].

This review paper aims to highlight recent progress in the field of plasma oncology, and will present LTPs as a promising tool for future focal cancer treatment. Comparisons are made between the mechanisms of existing therapies and how the properties of LTPs could lead to more favourable treatment outcomes. The prospect of combining LTPs with existing therapies and technologies to exploit potential synergies is outlined, as well as a speculative view suggesting how LTPs may be capable of overcoming treatment resistance. We also present novel data on the cytotoxic effect of LTP on cancer stem cells cultured directly from an aggressive prostate tumour. Finally, introduction of LTPs into clinical practice is evaluated, and the logistics of patient treatment is discussed.

## Low temperature plasmas as a source of reactive species

Mounting evidence in the scientific and medical literature suggests that LTPs rely strongly on the formation of reactive species to facilitate cellular responses. Processes such as ionisation, dissociation, excitation and recombination of atoms and molecules within the plasma lead to a chemically rich environment of reactive oxygen species (ROS) including atomic oxygen (O) [12, 13], hydroxyl (OH) [14], superoxide (O_2_^−^)[15], singlet-delta oxygen (^1^O_2_) [16] and hydrogen peroxide (H_2_O_2_) [17]. In addition, depending upon the gas composition and plasma geometry, reactive nitrogen species (RNS) may include atomic nitrogen (N) [18], nitric oxide (NO) [19], peryoxynitrite (ONOO^−^) [20] and other members of the NO_x_ family. The multitude of RONS generated by LTPs could provide significant advantages over other cancer therapies, e.g. radiotherapy and photodynamic therapy, which generally produce only ROS. Indeed, high concentrations of NO has been suggested to preferentially induce apoptosis in tumour cells, implying the action of nitrosative stress could prove crucial to successful cancer therapy [21].

The involvement of ROS in cancer initiation and progression [22], and their therapeutic potential [23] have been actively researched for many years. The cellular threat from low levels of ROS is well tolerated and neutralised through the action of enzymes including super oxide dismutase and catalase [24]. The inherent elevated metabolic activity in malignant cells (Warburg effect) may present a therapeutic window, as they are essentially already at their ROS-tolerance threshold or ‘red-line’ when compared with neighbouring normal cells [1, 25]. The creation of high levels of ROS is the mechanism by which long-established anti-tumour strategies, such as radio- [26] and some chemo-therapies [27, 28], operate to induce oxidative stress which result in cytopathic cellular responses. Given that LTPs create a multitude of reactive oxygen *and* nitrogen species (RONS) [29], they are an obvious candidate for cancer therapy; potentially being more efficacious than treatments which only involve ROS. This concept is discussed further in the context of treatment resistance in a later section.

**Fig. 2:**
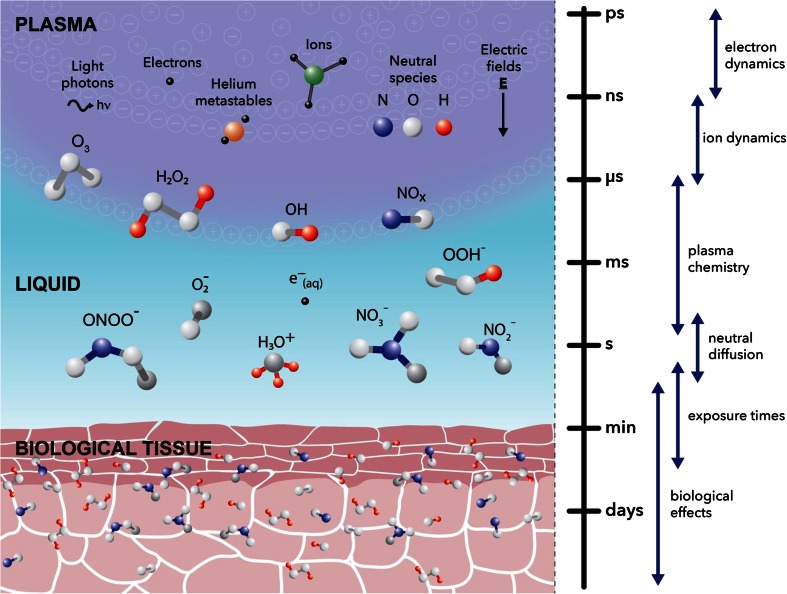
An illustrative representation of the multi-phase transfer of plasma species towards a biological sample. The main components of the plasma phase, including ions, photons and neutral species, are shown, leading to the creation of various RONS across the plasma-liquid interface and their propagation towards and diffusion through an arbitrary tissue layer. In addition, approximate timescales governing various phenomena across the plasma-liquid phases and biological interaction are outlined

The application of LTP to cells or tissues is a multi-phase process, which begins with an initial ignition and steady-state core plasma, followed by an afterglow plasma phase, leading to a diffusive interface with a liquid-like layer or environment. The liquid environment can either be represented by treatment of the cell culture media in laboratory experiments, or more physiologically the fluid within and surrounding a tumour in a clinical plasma application. This plasma-modified liquid environment then influences the cells and tissues around it. An illustrative overview of this process is depicted in Fig. 2, along with approximate time-scales for various phenomena in the plasma and liquid phases, and subsequent biological interaction.

The dynamics of the chemistry within the plasma core are extremely complex. Global models have been developed to capture this, which comprise in excess of 60 different species, involved in ∼1000 different reactions [2]. Translation to the liquid environment and ultimately a precise understanding of the specific extra- and intra-cellular RONS involved in both cellular effect and response, and their concentrations is vastly more so. Predictive numerical models have attempted to resolve and understand this complexity, including both the variation in chemistry between the gas-liquid-tissue phases [30], the fluxes of different reactive species at the tissue surface [31], and the influence of different molecular gas admixtures [13, 18, 32]. The mechanistic effects of LTPs on cells are presented in the following section.

## Mechanisms of LTP—cell interaction and response

LTPs create and transfer numerous RONS to the cellular environment, as discussed earlier. Current evidence implies that the production of RONS is primarily responsible for cytopathic effects of the plasma. However, other facets of LTPs may contribute to ultimate cell fate and treatment outcome.

LTPs have been applied to a range of different malignant cell lines in culture with extremely promising results. A range of common cellular responses have been documented including DNA damage [33, 34], decreased cell viability and clonogenicity [35, 36], reduced proliferation [37] and cell cycle arrest [38, 39]. From the growing literature, it would appear the cell death mechanism following LTP treatment varies with both the cell type and plasma source used. The vast majority of studies report apoptosis [19, 37, 40–43]; however, senescence [44] and non-apoptotic cell death [36] have also been presented. A summary of experimental approaches to cell treatment and subsequent cell death mechanism is given in Table 1. The studies presented therein were selected to reflect the different types of plasma, exposure times and approaches to treatment adopted within the field, and how these might relate to the observed outcomes. Elevated RONS levels are continually cited as the likely perpetrators of plasma-induced effects, leading to the activation of apoptotic pathways including TNF-ASK1 [46], ATM/p53 [19] and MAPK [15]. Furthermore, LTP effects have been shown to be (at least partially) alleviated by the use of various RONS scavengers [19, 47], further confirming the central role of reactive species produced by LTPs. Despite this, strong electric fields produced by some LTPs may play an important, synergistic role in plasma-cell interaction [48] and are discussed further in a later section.

**Table 1:** LTP treatment induces different paths to cell death. Summary of assorted cell treatment methods and associated death mechanisms for a range of malignancies

Cancer type	Method of treatment	Treatment duration	Cell death mechanism	Reference
Prostate cancer cell lines: PC-3 and LNCaP	In suspension, 500 μl volume	10 s	Apoptosis	Weiss et al. [37]
Glioma cell lines: U87, U373, A172	Adherent cells, 96-well plates, ∼40 % confluence	Up to 180 s	Apoptosis/necrosis	Siu et al. [40]
Lymphoma cell line: U937	Adherent cells, 10 cm plates, 5 ml volume	Up to 480 s	Apoptosis	Kaushik et al. [42]
Malignant cell lines from various sites	Adherent cells, 35 mm plates	30–60 s, up to 10 repeated exposures	Apoptosis	Ma et al. [19]
Colorectal cancer cell lines: Caco2, HCT116, SW480 and HT29	Adherent cells in various multi-well culture plates	Up to 30 s	Apoptosis	Ishaq et al. [41]
Glioma and colorectal cancer cell lines: U87MG-Luc2 and HCT-116-Luc2.	Adherent cells, 24-well plates, 500 μl volume	Up to 30 s	Apoptosis	Vandamme et al. [45]
Glioma xenografts: U87MG-Luc2	Subcutaneous tumours	6 min daily for 5 consecutive days	Apoptosis	
Head and neck cancer cell lines: FaDu, SNU1041, SNU899 and HN9	In suspension, 6 cm plates, 3 ml volume	1 s at either 2 or 4 kV	Apoptosis	Kang et al. [15]
FaDu xenografts	Subcutaneous tumours	20 s daily for 20 days	Apoptosis	
Various melanoma cell lines	Adherent cells, assorted culture plates, without culture medium	Up to 120 s	Senescence	Arndt et al. [44]
Prostate cancer primary epithelial cells	In suspension, 1.5 ml volume	Up to 600 s	Necrosis, autophagy	Hirst et al. [17]

Many investigators report a selective effect following LTP treatment, i.e. the plasma-effect preferentially targets tumour cells and leaves normal cells relatively unscathed [36, 49]. This is without doubt a highly desirable, ‘gold standard’ outcome. One explanation may be the rapidly dividing nature of tumour cells, increasing their vulnerability to DNA damage in M-phase [50], and/or the different tolerances of normal and cancer cells to elevated ROS levels [25] as alluded to earlier. The latter may explain a recent observation of an elevated autophagic response of normal cells when compared to tumour cells [17]. However, a more simple explanation may be the comparison of different cell types, for example normal fibroblasts with epithelial cancer cells, which may have quite different response profiles.

LTPs have also been applied to three-dimensional cell line models including spheroids and murine xenografts. Surface treatment of glioma xenografts with LTP showed a significant reduction in tumour volume, facilitated by ROS-induced caspase-3-dependent cell death [45]. In an earlier study, the same group showed that LTP treatment of tumours resulted in a 58 % increased lifespan over untreated mice [51]. Application of LTP in an in vivo head and neck cancer cell line model showed significant reduction in tumour mass and volume, verified by DNA fragmentation and caspase-3 positive staining, indicative of apoptosis through activation of p38 and JNK [15].

A recent study showed that primary prostate cells, cultured directly from patient tissue samples, rapidly underwent necrosis following exposure to LTP [17]. In addition, the effect on both normal and cancer prostate cells from the same patient was largely comparable. These findings imply that (a) primary cells may respond quite differently to LTP treatment than the broadly apoptotic response found in various cell lines, and (b) selective plasma effects may be less pronounced when LTP is applied to patients. Clearly, further verification of primary cells and primary xenografts from various tumour sites will provide further insight into patient response to LTP. The safe application of LTP to cancerous ulcers has been demonstrated for palliative purposes, but also showed partial tumour remission in some patients [52].

Direct and uniform exposure of all cells within a bulk tumour population to LTP treatment would be extremely technically challenging. However, it is conceivable that cell-to-cell communication will play a role in LTP treatment of a tumour. Radiation-induced bystander effects (RIBEs) are well documented following DNA damaging events and associated elevation in ROS levels in irradiated cells, which lead to extracellular stress-signalling to neighbouring non-irradiated cells [53]. Given that LTPs are known to inflict comparable initial cytotoxic effects on tumour cells, it would therefore seem logical to anticipate a similar plasma-induced bystander effect following LTP treatment [54].

Although much of the focus of plasma medicine studies centre around elevated ROS levels and their effects, the formation of strong localised electric fields by LTPs can also occur. These may interact directly with cell membranes and thus cause similar effects to those of emerging electroporative cancer therapies. Electroporation treatments utilise strong electric fields to irreversibly compromise cell membranes to provoke a cytocidal response. Nanoknife technology has been proposed for focal treatment of pancreatic [55], prostate [56] and renal cancers [57]. Numerical modelling has suggested that LTPs may create electric fields in the hundreds of kilovolt/centimetre (kV/cm) range [58], capable of penetrating a few cell layers, and generating sufficiently high fields within individual cells for electroporative effects [59]. The geometry and type of plasma will determine the presence and strength of the electric field. Novel methods and diagnostic techniques have quantified average field strengths of around 10–20 kV/cm within LTPs, but locally these may rise towards 100 kV/cm [60, 61]. Crucially, electric field strength has recently been determined for plasmas propagating through elongated capillaries [48]; the importance of which is discussed later. Electroporative effects have indeed been demonstrated biologically following plasma treatment [62], which may irreversibly damage cell membranes and aid the transfer of RONS into the cell, as well as permitting leakage of intra-cellular components. In some circumstances, plasmas can also generate focussed shockwaves that propagate through solutions (and into tissues), which have been shown to induce cell death in vivo [63].

## Combination of LTP treatment with existing cancer therapies to exploit synergistic gains

Whilst LTPs show clear potential to be an effective future cancer therapy in their own right, their efficacy could be further enhanced by combining them with existing treatment modalities. A recent study showed that a low temperature plasma gun was more effective than the chemotherapeutic agent gemcitabine in reducing both tumour volume and mass in an orthotopic pancreatic cancer model [64]. However, alternating plasma treatment with the drug saw further significant increases in treatment efficacy. This poses the possibility of combining plasma treatments with current standard treatment modalities, which may exploit potential additive or synergistic effects, leading to improved treatment outcomes.

LTPs may also be considered as an alternative option to treat malignancies that are resistant to the conventional treatment approaches. One example is temozolomide (TMZ), the standard initial chemotherapeutic agent prescribed to glioblastoma patients. However, tumours which express high levels of the enzyme O6-methylguanine-DNA methyltransferase (MGMT) show high resistance to TMZ [65]. When treated with LTP, glioblastoma cell lines (including MGMT-positive cells) showed reduced viability and clonogenicity, cell-cycle arrest, and ultimately apoptosis far in excess of TMZ-treated control cells [66]. A similar finding was observed in a chemo-resistant hepatocarcinoma model, where treatment with LTP lead to significant cytotoxic effects [67]. This demonstrates the potential for the use of LTPs as a salvage treatment option for patients who have failed the standard treatment approach, or perhaps pre-emptively in tumours that are known to be resistant to certain agents.

Recent studies have demonstrated the use of gold nanoparticles (AuNPs) for targeted delivery into tumour cells as drug carriers [68] or radiosensitisers [69]. AuNPs may also provide a means to effectively target cancer stem cells (CSCs) [70], a small population of cells believed by many to be the root of treatment resistance and recurrence, which is discussed further in the following section. The potential of AuNPs has led plasma physicists to investigate their use in conjunction with LTPs [71]. When utilised together, the combination AuNPs with LTP treatment enhanced efficacy beyond that of either agent alone in glioblastoma cells [72]. Treatment with LTP may also increased the uptake of AuNPs into malignant cells [73]. The amalgamation of LTPs and AuNPs may also present an opportunity to increase the cytotoxic selectivity of LTP towards tumour cells [74, 75].

The exact mechanism of plasma-induced cytopathic effects could prove crucial to the long-term success of any prospective anti-cancer treatment, broadly speaking: apoptosis or necrosis. Apoptotic cell death is potentially immunosuppressive and thus can assist immune system evasion of the tumour [76, 77]. However, in several pre-clinical studies addressing the combination of radio- and immuno-therapies to improve therapeutic potential [78], it has been shown that necrotic cell death can increase tumour immunogenicity through induction of heat shock protein expression [79]. Moreover, necrosis is induced by thermally ablative treatments such as cryotherapy [80], radiofrequency ablation [81] and HIFU [82], and is known to cause local inflammation at the treatment site. As mentioned previously, it has recently been demonstrated that prostate cancer cells cultured directly from patient tissue samples and treated with LTP rapidly initiate necrotic cell death [17]. This speculatively raises the question of immune activation against the tumour following plasma application, and the possibility of spontaneous regression of metastatic tumours, as has been occasionally recorded following radiotherapy [83], radiofrequency ablation [84, 85] and cryotherapy [86]. Direct combination with immunotherapy may present further synergistic prospects [87]. As a result, it may be argued that plasma-induced cell death via necrosis *could* provide the most effective long-term treatment outcome. Should this be the case, immune checkpoint inhibitors (such as nivolumab, which has very recently demonstrated efficacy in the treatment of advanced nonsquamous non-small-cell lung cancer and metastatic melanoma [88, 89]) may present an interesting prospect for future use in conjunction with LTP to boost tumour immunogenicity. Another thought-provoking concept is the direct stimulation of immune cells with LTPs, potentially increasing the efficacy of macrophages against tumour cells [90].

## Overcoming resistance to conventional treatments with low temperature plasmas

As with any prospective new treatment, there are questions regarding potential treatment resistance, as commonly experienced with some currently applied cancer therapies. Tumour hypoxia has been identified as one probable factor in radio- and chemo-therapeutic resistance and tumour invasiveness [91]. Supporting evidence has been reported recently in many different malignancies including those of the lung [92], liver [93], breast [94, 95] and brain [96]. Whilst direct DNA damage is inflicted by energetic particles, secondary damage following radiotherapy is caused by the production of oxygen radicals from the interaction of ionising X-rays and molecular O_2_ in tissues and the local environment. As a result, in oxygen-deficient regions of the tumour, lethal DNA damage may not be achieved [97]. Hypoxia may not be so much of an issue for LTP therapy, since the majority of LTP cancer studies feed small admixtures of molecular oxygen (or nitrogen) into the main gas flow to aid the production of oxidative (and nitrosative) radicals. As such, LTP treatment could provide oxygen radicals directly to the treatment site, circumventing the need for endogenous O_2_ in the tissue (as with radiotherapy), which may surmount the issue of hypoxic resistance. The success of this theory would depend strongly on the means of treatment administration and reactive species penetration, which are discussed in the subsequent section.

CSCs have been proposed to be the root of both disease initiation [98] and recurrence [99]. They have been widely implicated in both radio- and chemo-resistance [100–103]. One reason for this may be higher levels of heterochromatin in CSCs compared to the bulk population, affording added protection against DNA damaging treatments [104]. It is also thought that CSCs have higher levels of ROS-quenching enzymes in order to alleviate toxicity effects from reactive species formation [105] more effectively than their differentiated counterparts. Overloading CSCs with an abundance of RONS generated by LTPs may overcome this protective shield.

**Fig. 3:**
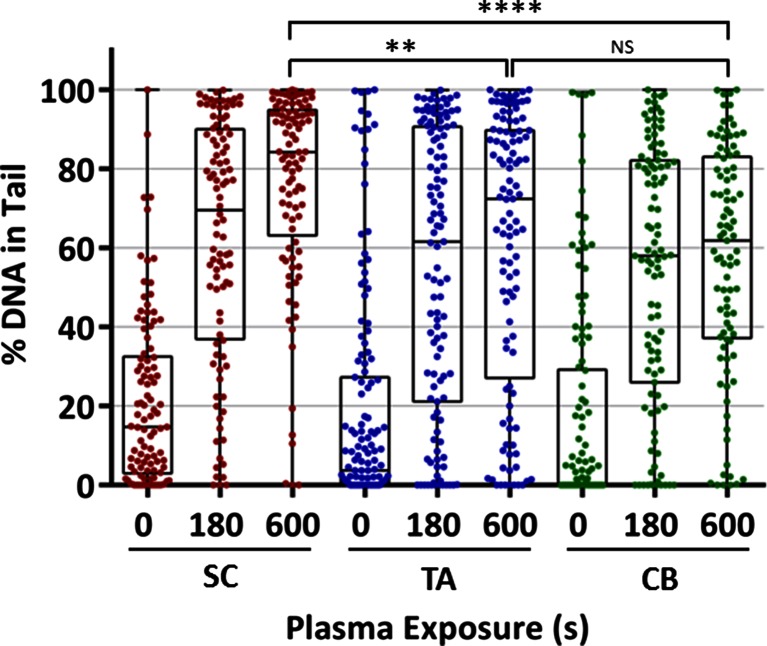
LTP induces DNA damage in cancer stem cells. Prostate cancer stem cells (*SC*), transit amplifying (*TA*) and committed basal (*CB*) cells were cultured and fractionated [106, 107] from a Gleason grade 9 metastatic tumour, and treated as described in Hirst et al. [17]. Statistical analysis of plasma treatments was calculated using Mann–Whitney test against untreated samples and showed *P* < 0.0001 significance, unless otherwise indicated (**P < 0.01, ****P < 0.0001)

Our own experimental evidence suggests that LTP can be delivered in cytotoxic doses to CSCs. Figure 3 shows high levels of DNA damage (quantified using the Comet assay, based on [108]) following LTP jet treatment [17], irrespective of cellular sub-population. Here, the cells treated with plasma were primary prostate epithelial cells, cultured directly from an aggressive Gleason grade 9 tumour. The cells were sorted into sub-populations [106] and treated in suspension. Whilst this is very preliminary data, its inclusion serves to demonstrate the potential of LTP to induce highly significant cytotoxic effects in cells that are thought to be a causal factor in treatment resistance and relapse.

## Progress towards the clinical use of low temperature plasmas

Many different plasma designs and geometries exist across academic institutions, e.g. [6, 7], demonstrating a broad versatility, but also highlighting the fact that direct data comparison can be problematic. Standardised ‘reference’ plasma jets are being developed across research centres to help unravel the fundamental plasma physics and chemistry. It is also possible that different LTP sources will ultimately find different clinical uses. Some proposed uses include intra-operative treatment of potentially positive surgical margins following tumour excision by surgery [37], injection of plasma-activated media into the tumour [109, 110], and decontamination of ulcerations in advanced head and neck cancer patients as mentioned earlier [52].

The majority of published studies in the field of plasma oncology focus on the direct application of LTPs to tumour cells. To fully eradicate solid tumours, the cytotoxic effect of plasma application must be capable of penetrating several layers of cells. A recent study on colorectal cancer cells, cultured as spheroids in suspension and treated with an LTP jet, showed a reduction in growth rate at low exposures and a complete growth arrest at longer plasma exposures of [111]. However, only the first few outer layers of the spheroid showed γH2AX-positive foci, suggesting that plasma-induced cell damage was surface-limited. Another report used agarose gel as a tissue-substitute to model the transfer of RONS across a biologically relevant interface. Reactive species were detected in the liquid regardless of whether the plasma jet was in direct contact with the agarose or not, and even after the plasma had extinguished [112]. This suggested that RONS were released from the agarose, created in the liquid environment as secondary reactions, or both, even after treatment. This simple model shows that reactive species produced by LTPs can cross a tissue-like interface (at least up to a few mm), which when combined with a potential plasma-induced bystander effect gives hope for cytopathic plasma-effects in solid tumours. Despite several in vivo studies showing promising levels of tumour reduction following LTP application [15, 45, 113], complete tumour eradication and long-term disease-free outcome remains to be proven.

**Fig. 4:**
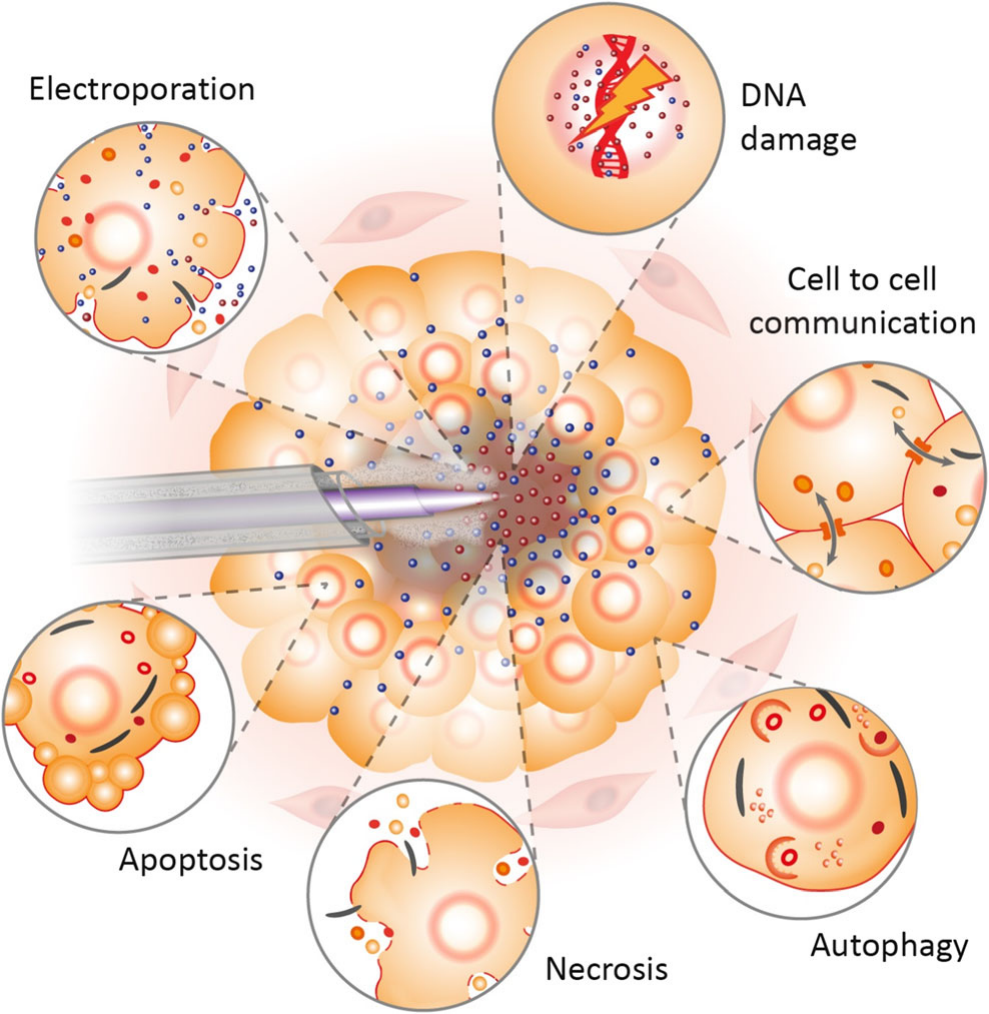
Illustration of LTP treatment of a tumour. In the proposed approach, the LTP probe is inserted under needle guidance into the core of the tumour. The plasma is then ignited, creating short-lived reactive species (*red dots*) that induce DNA damage, necrosis and potentially electroporative effects to cells in the immediate vicinity. The diffusion of longer-lived species (*blue dots*) to the tumour periphery is shown, contributing to apoptotic and plasma-induced bystander effects. Proposed cellular effects and responses are estimated based on their proximity to the plasma source. Gas extraction is also indicated through a co-axial configuration in the LTP probe. Elements of this figure are adapted from Hirst et al. [17]

The most successful method of realising an effective, focal and minimally invasive surgical approach is likely to be penetration of the plasma into the tumour core, to destroy the cancer radially outwards. Although many tumours are multi-focal, it has been argued that targeted treatment to only the index lesion of a localised tumour is sufficient to provide satisfactory disease control [114], in addition to limiting treatment invasiveness. The concept of inserting the plasma transperineally into the centre of a prostate tumour was proposed in a recent review article [6], and is expanded for an arbitrary solid malignancy in Fig. 4. We propose that this concept should ensure enhanced targeted treatment of a tumour, compared to conventional surgical or radiotherapy techniques, and more controlled tumour volume destruction than is feasible with alternative ablative techniques such as RFA or cryotherapy.

Assuming the effects of LTP could propagate beyond a few cell layers (be it directly or via bystander effects), precisely monitored plasma ablation should also enable a satisfactory clearance zone to be achieved. This implies that damage to normal cells is not necessarily a negative feature, as a degree of collateral damage is a more favourable consequence than incomplete tumour ablation. Clearance margins of ∼1 cm have been suggested in some cases [115], to maximise long-term disease-free outcome.

The propagation of LTPs in liquid environments has been demonstrated experimentally [116], where, depending on the operating parameters, the plasma may adopt either a bush- or tree-like formation after generation [117]. Clearly, the degree of relative moisture within the tumour environment will play a role in the plasma propagation and chemistry, and is likely to vary from tumour to tumour. Delivery of the plasma to areas that are potentially difficult to access and penetration inside the tumour are two of the main technical hurdles with this proposition; nevertheless, evidence within the literature suggests both can be overcome. As plasmas can be propagated along tubes of metres in length [118], precise LTP delivery even to tumours deep within the body should be possible in principle. In shorter tubes, plasmas have been sustained in tubes as small as ∼10 μm in diameter [119]. Internal plasma application has already been evaluated as effective and well-tolerated in a pancreatic in vivo model [113]. As some internal applications may require longer tubing lengths than others, the inherently short lifetimes of the most reactive (and thus most damaging) species may curtail their journey from source to target. However, provided an active plasma emerges from the end of the tube where electrons are present, short-lived species will be created locally at the application site. This concept is illustrated in Fig. 4, but largely depends on the plasma source used. Regardless, a rigorous knowledge of the RONS densities emerging from the specific aperture used for application is essential. It has recently been suggested that control and selectivity towards different reactive species may be achievable by using different feed gases [120]. Maximal lethality of treatment is likely to be found by tuning the plasma operating conditions including voltage waveform parameters, gas composition and treatment duration [121]. Finally, some form of gas flow extraction (as highlighted in Fig. 4) during LTP treatment would almost certainly be necessary to minimise the risk of embolisms, and could be combined with cyclic LTP application.

## Conclusions

Earlier diagnosis and accurate targeting, combined with minimal damage to surrounding tissues and reduced patient side effects, has led to increased popularity of tumour treatment with thermal and non-thermal ablative focal therapies. Over the last decade, LTPs have demonstrated their potential as a novel approach in the targeted treatment of cancer. Both in vitro and in vivo studies have shown promising results in a wide range of different malignancies. In addition, both modelling and experimental studies are beginning to unravel the complex interplay of plasma-liquid-cell interphases. Through precise application and accurate monitoring, LTPs could offer defined and effective treatment for many tumours, whilst minimising side effects to the patient. This review has highlighted the multifaceted action of LTPs, through the formation of a rich chemistry containing RONS and the possible contribution of strong electric fields in biological response. It has also speculatively outlined the potential for the application of LTPs as a combination therapy in conjunction with other current approaches, and how they may be able to overcome treatment resistance. Finally, a plausible treatment approach is presented, demonstrating how LTPs might be applied to any arbitrary solid mass, to achieve maximum lethality to the target lesion. It is hoped that the evidence and concepts presented in this paper have conveyed the undeniable promise of LTP technology for the future treatment of cancer.
